# Neurocirculatory Myasthenia

**Published:** 1919

**Authors:** 


***Neurocirculatory Myasthenia.*** By ANDREW MCFARLANE (Albany, N.Y.), Camp Gordon, Atlanta, Ga. *Journal A. M. A.*, Aug. 31, 1918.

That the sub-standard soldier is a fact detrimental to army efficiency is pointed out by the author. Therefore, one of the first steps in making a new army is to sort out those who are manifestly unfit, and to a certain extent this is effected. But as the training becomes more intense, soldiers are reported by their regimental surgeons as having defects that are serious, but not so obvious as to have caused their earlier rejection, and so far as they are concerned make the routine examination relatively worthless. McFarlane reports the results of later examinations of such cases at Camp Sevier, S. C. A cardiovascular detachment of ten tents was opened, and of forty-one patients referred to it, seven were found to have organic disease of the heart, and were referred to the disability board for discharge. Five were adjudged free from cardiovascular disability and returned to the command with a request for their further observation. "The remaining twenty-nine soldiers all had this in common: they were reported as inefficient soldiers, though no determinable organic cardiovascular lesion was found. They presented practically the same syndrome: marked cyanosis of the hands extending up above the wrists; coldness and sweating of the hands and feet; sweating under the armpits, even when in a cold tent; tremor; flushing; marked lability of the pulse and blood pressure on standing or lying down, and after exercise; marked thrill, often felt over the precordium; no increase of cardiac dulness; booming first sound or indefinite murmur at the apex; frequent accentuation of the second pulmonic sound, and mental instability. After exercise these patients became more or less breathless and cyanotic, and complained of precordial pain, palpitation, giddiness and abnormal exhaustion." Full details of the defects are given in tabulated form, and also the results of examinations according to Pignet's formula. Only one of these men could be rated as strong, and examinations by nose and throat specialists revealed apparent foci in all. The British experience in this regard is referred to, but their problem was somewhat different as the soldiers had already been to the front and were returned for inefficiency. In the great majority of cases the recognition of this sub-standard quality was not difficult. Similar conditions were noticed in the Civil War by DaCosta. In the British service the aim has been to build these men up with exercises, and similar methods were employed here. They were sent to the base hospital clinics for treatment of their teeth and tonsils, and graduated exercises were given each day, with graded hikes in the afternoon, all exercises being overseen by two physicians. The problem was to determine what each soldier could do with his own physique, irrespective of height and weight. The scientifically graduated exercises were followed later by the graduated work of a soldier. Games and proper occupations were encouraged. In this way, by building up the system and training, the weakened muscles of some good soldiers could be saved to the country. Others might be employed for special service, and the remainder returned to civil life better and wiser for their army experience.

